# Histone deacetylase 3 indirectly modulates tubulin acetylation

**DOI:** 10.1042/BJ20150660

**Published:** 2015-11-27

**Authors:** Travis Bacon, Caroline Seiler, Marcin Wolny, Ruth Hughes, Peter Watson, John Schwabe, Ronald Grigg, Michelle Peckham

**Affiliations:** * School of Molecular and Cellular Biology, Faculty of Biological Sciences, University of Leeds, Leeds LS2 9JT, U.K.; † Department of Molecular and Cell Biology, Henry Wellcome Laboratories of Structural Biology, University of Leicester, Lancaster Road, Leicester LE1 9HN, U.K.; ‡ School of Chemistry, Faculty of Maths and Physical Sciences, University of Leeds, Leeds LS2 9JT, U.K.

**Keywords:** acetylation, histone deacetylase 3, microtubules, tubulin

## Abstract

Histone deacetylase 3 (HDAC3), a member of the Class I subfamily of HDACs, is found in both the nucleus and the cytoplasm. Its roles in the nucleus have been well characterized, but its cytoplasmic roles are still not elucidated fully. We found that blocking HDAC3 activity using MI192, a compound specific for HDAC3, modulated tubulin acetylation in the human prostate cancer cell line PC3. A brief 1 h treatment of PC3 cells with MI192 significantly increased levels of tubulin acetylation and ablated the dynamic behaviour of microtubules in live cells. siRNA-mediated knockdown (KD) of HDAC3 in PC3 cells, significantly increased levels of tubulin acetylation, and overexpression reduced it. However, the active HDAC3–silencing mediator of retinoic and thyroid receptors (SMRT)–deacetylase-activating domain (DAD) complex did not directly deacetylate tubulin *in vitro*. These data suggest that HDAC3 indirectly modulates tubulin acetylation.

## Abstract (precis)

Histone deacetylase 3 removes acetyl groups from lysine residues, thereby modifying protein function. It is found in both the nucleus and the cytoplasm. We have discovered that it can indirectly deacetylate tubulin, a cytoplasmic protein that forms microtubules, thus modifying the microtubule.

## INTRODUCTION

Histone deacetylases (HDACs) comprise a family of enzymes that remove acetyl groups from proteins, with the first family members isolated over 40 years ago [1]. Although these enzymes were first found to deacetylate nuclear histone proteins, hence the name ‘histone’ deacetylase [2], more recently HDACs have also been shown to act on non-histone proteins including cytoplasmic proteins in some cases [3]. In mammals, four classes of HDAC exist [4,5], with classification based on sequence homology to the original yeast enzymes and domain organization. Class I HDACs include HDAC1–HDAC3 and HDAC8, and, with the exception of HDAC3, localize primarily to the nucleus, although HDAC8 additionally localizes to the cytoplasm in smooth muscle cells [6]. Class II HDACs can be divided into two subclasses: IIa (HDAC4, HDAC5, HDAC7 and HDAC9), which are found in both the nucleus and the cytoplasm and IIb (HDAC6 and HDAC10), which are primarily cytoplasmic in their localization. Of the Class III HDACs (sirtuins, SIRT1–SIRT7) SIRT1, SIRT6 and SIRT7 localize to the nucleus, SIRT2 to the cytoplasm and SIRT3, SIRT4 and SIRT5 to mitochondria, with SIRT3 also localizing to the nucleus. The sole member of the Class IV family (HDAC11) is found in both the nucleus and the cytoplasm.

The functions of HDAC3 in the nucleus have been well described. For example, HDAC3 forms a well-characterized protein complex with the nuclear receptor co-repressor (NCoR) or the homologous SMRT (silencing mediator of retinoic and thyroid receptors) complex in the nucleus [7] where it interacts with a conserved deacetylase-activating domain (DAD) within NCoR/SMRT, which activates the complex [8–10]. The complex is recruited to specific promoters, where HDAC3 deacetylates the histones, leading to gene silencing. This complex and HDAC3 have been shown to be essential for maintenance of chromatin structure and genome stability [11]. Ins(1,4,5,6)*P*_4_ regulates the interaction of HDAC3 with SMRT and has been suggested to regulate other HDAC3–protein complexes [12].

HDAC3 is involved in a wide range of other interactions that are less well characterized. It can interact with Class II HDACs (HDAC4, HDAC5, HDAC7 and HDAC9) via the transcriptional co-repressor NCoR/SMRT ([13,14] and reviewed in [15]). It can bind to, and be activated by, the nuclear envelope protein emerin [16], which regulates expression and localization of muscle transcription factors during myogenesis [17]. It can bind to and deacetylate nuclear factor-κB (NF-κB), playing a role in interleukin-1 (IL-1) expression and the inflammatory response [18]. Normal huntingtin protein binds to and sequesters HDAC3 primarily in the nucleus [19], but mutant huntingtin does not, exposing neurons to HDAC3’s neurotoxic effect [20]. HDAC3 also binds to the ataxin–1NCoR–SMRT complex and plays a role in other polyglutamine-repeat diseases such as spinocerebellar ataxia (reviewed in [15]).

In addition to its nuclear roles, HDAC3 has roles in the cytoplasm, as it is found in both the cytoplasm and the nucleus and can shuttle between them [21]. Although the nuclear roles of HDAC3 have been well established, its cytoplasmic roles are less well characterized, whereas roles in signal transduction, and particularly in inflammatory signalling, have been established. For example, HDAC3 binds to IκBα (inhibitor of NF-κBα) in the cytoplasm, and breakdown of IκBα following stimulation by tumour necrosis factor-α (TNF-α) results in the nuclear translocation of HDAC3 [22]. Conversely, deacetylation of RelA (p65) by HDAC3 in the nucleus, allows the nuclear export of the NF-κB complex [23]. Its ability to bind to and deacetylate members of the signal transducer and activator of transcription (STAT) proteins STAT1 [24] and STAT3 [25] may play a role in the cytoplasmic retention of these proteins. HDAC3 can localize to the plasma membrane in a subpopulation of cells, where it forms a complex with c-Src, implicating c-Src (a tyrosine kinase) in HDAC3 regulation [21]. HDAC3 has been shown to localize to muscle sarcomeres, and deacetylate the myosin heavy chain [26]. Finally, in mitosis, a complex of HDAC3, NCoR1, transducing-β-like protein 1 (TBL1) and TBL1-related proteins has been reported to localize to and maintain the structure of the mitotic spindle [27].

Selectively inhibiting HDAC3 has been suggested to hold promise in treating a range of diseases, from inflammation, to neuro-protective effects [15] and cancer therapy [28]. Several specific HDAC3 inhibitors have been described (reviewed in [15]). One of these, MI192, is highly selective for HDAC3, and has been shown to inhibit TNF and IL-6 production in peripheral blood mononuclear cells [29] as well as promote apoptosis in acute myeloid leukaemic cell lines [30]. In common with other Class I HDAC selective inhibitors, such as MGCD0103, which is selective for both HDAC1 and HDAC2, but 30-fold less active against HDAC3, and MS-275, which is highly active against HDAC1, but much less active against HDAC2 and HDAC3 [14], MI192 has the same terminal benzamide structure that chelates the Zn^2+^ ion at the HDAC active site. MI192 is less effective at inhibiting HDAC3 than trichostatin A (TSA), but it is selective for Class I HDACS, and has a greatly enhanced activity against HDAC3 compared with HDAC1 within this subgroup. Thus we speculated that this HDAC3 inhibitor should be useful in probing for its other, as yet uncharacterized, cytoplasmic roles. In particular, we were interested in determining whether HDAC3 was able to modulate α-tubulin acetylation, in common with two other HDACs found in the cytoplasm, HDAC6 and SIRT2 [10,31–33]. We followed up our findings with the inhibitor with siRNA-mediated knockdown (KD) and overexpression experiments. Taken together our results suggest that HDAC3 may also be able to modulate α-tubulin acetylation as one of its cytoplasmic roles.

## MATERIALS AND METHODS

### Cell lines, antibodies and expression constructs

Immortalized human prostate cancer (PC3) cells, obtained from the A.T.C.C. (Manassas, VA, U.S.A.), were used in these experiments. They were cultured in RPMI 1640 medium containing GlutaMAX™ (Gibco), supplemented with 10% heat-inactivated FBS, 1% penicillin and 1% streptomycin. Antibodies used in staining and/or Western blotting were: anti-acetylated α-tubulin (Sigma), anti-α-tubulin (Serotec), anti-HDAC3 (rabbit, Santa Cruz Biotechnology), anti-HDAC6 (rabbit, Santa Cruz Biotechnology) and anti-total extracellular-signal-regulated kinase (ERK; p44/42 mitogen-activated protein kinase, Cell Signaling Technology). Secondary antibodies were used at the following dilutions: anti-mouse, 1:250 [Alexa Fluor 647 IgG (H+L), Molecular Probes, A21235]; anti-rat, 1:250 [Alexa Fluor 488 IgG (H+L), Molecular Probes, A21208]. Additional markers used were 546 phalloidin (actin, 1:100 dilution), DAPI (nuclear, 1:500 dilution). Note that, although we routinely used anti-total ERK as our loading control in the data shown here, reprobing the same blots with glyceraldehyde-3-phosphate dehydrogenase (GAPDH) showed similar results, and checks on the equivalent SDS gels, also confirmed similar protein loading on each of our gels and blots.

Human HDAC3 clones were obtained from Professor Edward Seto [34]. The HDAC3–GST bacterial expression construct was obtained from Professor Edward Seto [34], and subcloned into pEGFPC1 to generate a GFP-fusion protein. The entire eGFP–HDAC3 cDNA was then excised and subcloned into the pDC315 adenoviral plasmid (Microbix). This plasmid was used to make adenovirus as described in [35]. To infect cells, 1 μl of purified virus was diluted in 500 μl of RPMI 1640 medium before slowly adding to the cells (multiplicity of infection of ∼10) and incubating for 24 h at 37°C with 5% CO_2_.

### Immunostaining

Cells were grown on glass coverslips, fixed with 2% paraformaldehyde in PBS and stained using standard procedures [36]. Cells were imaged using a Deltavision Deconvolution microscope or a Zeiss 880 Confocal with AiryScan (Figure 5). Images used for quantification (Figure 5) were captured using the Deltavision system, using the same exposure settings. The fluorescence intensity of a 75 pixels×75 pixels square was measured using ImageJ (NIH), for nine cells, from at least two separate experiments. Images for mitotic cells were captured using the Deconvolution microscope using the ×100 objective, 1×1 binning and a stack size of 20 over a total of 4 μm. The size of the spindles and the area was measured from the final maximum intensity projection image for the total stack, using ImageJ.

### HDAC3 inhibitor

The cells were treated with the HDAC3-selective inhibitor MI192 at a range of concentrations, for 0.5–24 h. MI192 was produced by Ronald Griggs (Department of Chemistry, University of Leeds) and tested using MS and micro-analyses to confirm its purity. MI192 was solubilized in DMSO (Sigma), and all MI192 concentrations in the experiments described here were delivered in 0.1% DMSO. Control cells were treated with 0.1% DMSO only.

### HDAC activity assay

A colorimetric HDAC activity assay kit (BioVision) was used to assess HDAC activity in protein samples extracted from treated PC3 cells. Sample protein (50 μg) was incubated with a colorimetric acetylated lysine-containing substrate for 1 h at 37°C in 5% CO_2_. Lysine developer was then added, followed by an additional 30-min incubation. The deacetylated lysine residues react with the lysine developer, releasing a chromophore from the substrate, which was measured spectroscopically at 405 nm using a colorimetric plate reader (POLARstar, Optima).

### Western blotting

Cells were lysed (30 min, 4°C) in lysis buffer [150 mM NaCl, 0.05 M Tris/HCl (pH 8), 1% Triton X-100, 1 mM EDTA (pH 8) with protease inhibitor cocktail (Thermo Scientific)]. Lysates were clarified by centrifugation, protein content quantified by BCA assay, then samples mixed with 2 × Laemmli buffer for use in protein gels and blots (30 μg was added to each well). If required, membranes were stripped using Restore Western Blot Stripping Buffer (Thermo Scientific) and then re-probed. ImageJ was used to quantify protein expression from the Western blots. ERK1/2 was used as a loading control in all the blots, to compensate for any differences in protein loading.

### RNAi

siGENOME SMARTpool siRNA (GE Healthcare Dharmacon) for HDAC3 and HDAC6 was used to KD their expression. Cells were seeded at a density of 30 000 cells/ml in growth medium, allowed to adhere and grow overnight. Lipofectamine® RNAiMAX Reagent (Invitrogen, Life Technologies) was used for transfections. Maximum KD (>80%) was achieved after 72 h.

### Microtubule dynamics

To visualize microtubule dynamics in live cells, cells were infected with an adenovirus for eGFP–end-binding 1 (EB1), and cells were imaged by time-lapse imaging on a Deltavision deconvolution microscope, fitted with an incubator set to a temperature of 37°C, and using the ×63 oil objective. Prior to imaging, cells were treated for 1 h with increasing concentrations of MI192. Images of eGFP–EB1-labelled microtubule tips were taken every 2 s, and movement of the EB1 fluorescent spots were subsequently tracked using the MTrackJ plug-in in ImageJ.

### Flow cytometry

MI192 cytotoxicity was assessed with flow cytometry using an annexin V and propidium iodide Apoptosis Detection Kit (BD Pharmingen) to quantify the proportion of healthy, apoptotic and necrotic cells following 24 h of control (0.1% DMSO) or maximal (10 μM) M192 treatment.

### HDAC3–SMRT 350–480 complex deacetylation activity

The HDAC3–SMRT 350–480 complex was expressed and purified from human embryonic kidney (HEK)-293 cells as described in [37]. HDAC activity assays were carried out in a black 96-well plate. In a final volume of 50 μl, 50 nM HDAC3–SMRT 350–480 [with or without 100 μM suberoylanilide hydroxamic acid (SAHA) as required] was incubated with 100 μM t-butoxycarbonyl-lysine-7-amino-4-methylcoumarin (BOC-Lys-AMC) substrate for 30 min at 37°C in 50 mM Tris/HCl, pH 7.5, and 50 mM NaCl. Reactions were developed by the addition of 50 μl of 2 mM trichostatin A, 10 mg/ml trypsin, 50 mM Tris/HCl, pH 7.5, and 100 mM NaCl. Fluorescence was then measured at 335/460 nm using a Victor X5 plate reader (PerkinElmer).

Tubulin was polymerized in general tubulin buffer (80 mM PIPES, pH 7.0, 2 mM MgCl and 0.5 mM EGTA) plus 1 mM GTP and 20 μM taxol at 35°C for 20 min. HDAC3–SMRT 350–480 complex (11.3 μM) was incubated with 0.2 nM microtubules for up to 2 h at 37°C. Solution containing microtubules only was used as a control. Samples were collected every 0.5 h and immediately mixed with Laemmli buffer. Samples were subjected to SDS/PAGE and transferred on to a nitrocellulose membrane. Anti-acetylated microtubule antibody was used to assess deacetylation efficiency and anti-β-tubulin antibody was used as a loading control.

### Microtubule-binding assay

A microtubule-binding assay was carried out using the Microtubule Binding Protein Spin-down Assay Kit (Cytoskeleton), following the recommended protocol. Microtubule-associated protein and BSA were used as a positive and negative controls respectively. Amounts of 2 μg (0.6 μM) and 5 μg (1.6 μM) of the HDAC3–SMRT complex were used as test samples. Samples were incubated at room temperature for 30 min and spun down at 100000 ***g*** for 40 min at room temperature. Binding of control proteins was assessed by SDS/PAGE, whereas binding of HDAC3, was assessed by Western blotting using anti-HDAC3 antibodies, as it is similar in size to tubulin.

### Data and statistical analysis

Microsoft Excel was used to quantify sample protein concentrations and HDAC activity, as well as EB1 trafficking speed, before statistical analyses were performed on GraphPad Prism 6. ImageJ was used to analyse immunoblots and digitized images of immunostained cells. GraphPad Prism 5.0 was used to prepare graphs and analyse data. Data are presented as means ± S.E.M., for at least three separate experiments (*n* ≥ 3). A two-way ANOVA was used to compare differences between groups and statistical significance was accepted for *P*≤ 0.05.

## RESULTS

### MI192 inhibits HDAC3 and alters tubulin acetylation in PC3 cells

**Figure 1:**
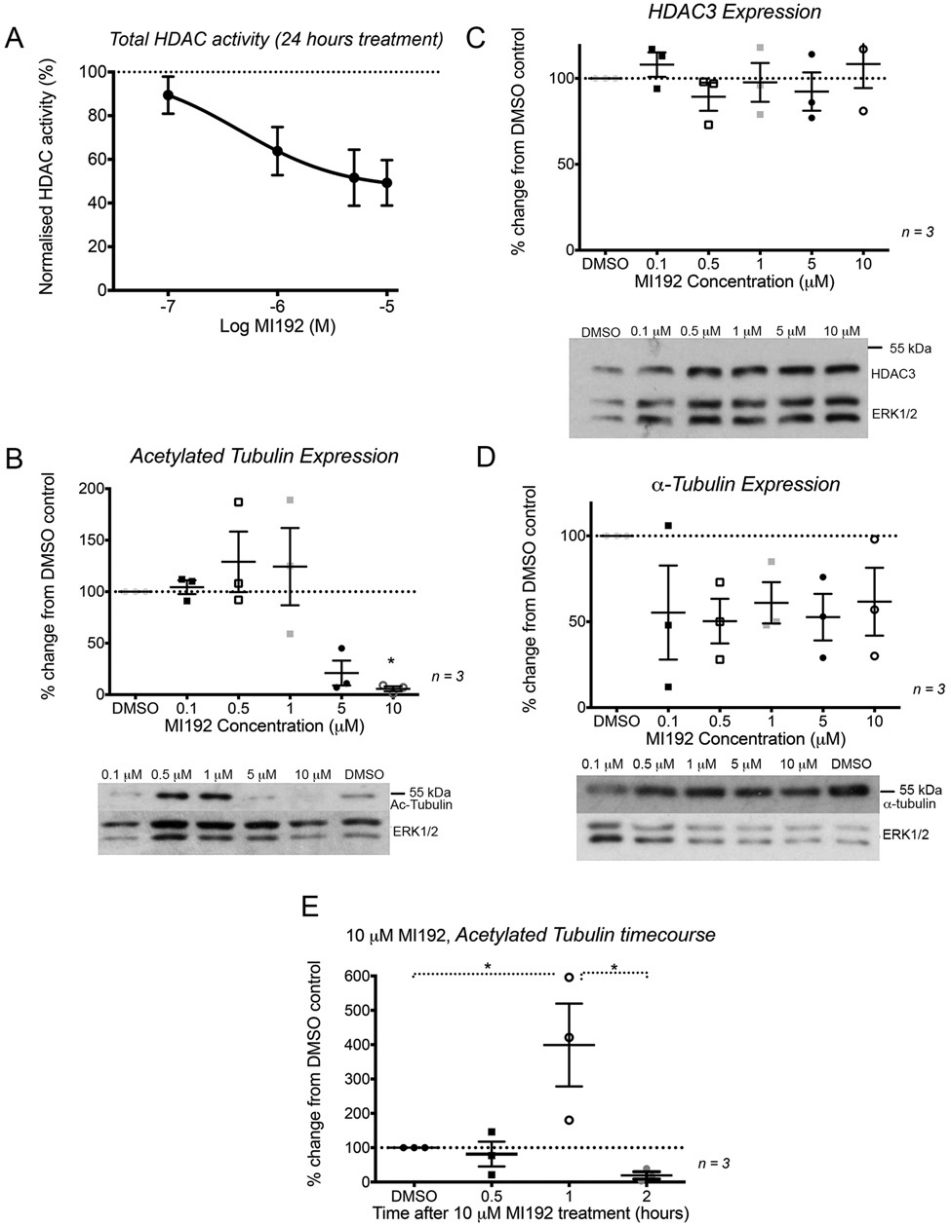
MI192 inhibits HDAC3 activity and alters tubulin acetylation levels after 24 h of treatment (**A**) The activity of HDAC measured from cell extracts. (**B**–**D**) Graphs and representative Western blots showing the effect of treating PC3 cells with a range of MI192 concentrations for 24 h on the levels of acetylated tubulin. (**E**) Changes in levels of acetylated tubulin after treatment of cells with 10 μM MI192 for 0.5–2 h was analysed by Western blotting. Data are shown as individual points, with means ± S.E.M. superimposed. *P< 0.05 (*n*=3 experiments).

MI192 is able to inhibit HDAC3 activity in PC3 cells, as shown by an HDAC activity assay (Figure 1A). MI192 reduced total HDAC activity by 49±9% at a concentration of 10 μM after 24 h of treatment, with an IC_50_ value of 0.45 μM. This value is similar to that measured previously for HeLa cell nuclear extracts [30].

MI192 inhibition of HDAC3 had a complex effect on tubulin acetylation levels. Continued treatment of PC3 cells for 24 h at low (0.5 or 1 μM) MI192 concentrations slightly increased tubulin acetylation levels, but at higher concentrations (5 or 10 μM), levels of tubulin acetylation decreased. This decrease was significantly different from control cells for 10 μM MI192 (Figure 1B). These effects were not due to changes in HDAC3 expression levels (Figure 1C). Moreover, total levels of α-tubulin did not change (Figure 1D). In contrast, treating PC3 cells for just 1 h with 10 μM MI192 significantly increased the levels of acetylated tubulin (Figure 1E). The levels of acetylated tubulin were 4-fold higher than those in control cells at the same time point (399±98%, *P*≤ 0.05) (Figure 1E). After treating cells for 2 h with 10 μM MI192, levels of acetylated tubulin had already begun to decrease to below the levels found in untreated cells (20±9%) (Figure 1E). Thus, treatment with 10 μM MI192 results in an acute increase in tubulin acetylation levels at 1 h, followed by a rapid fall which is maintained over the next 24 h.

**Figure 2:**
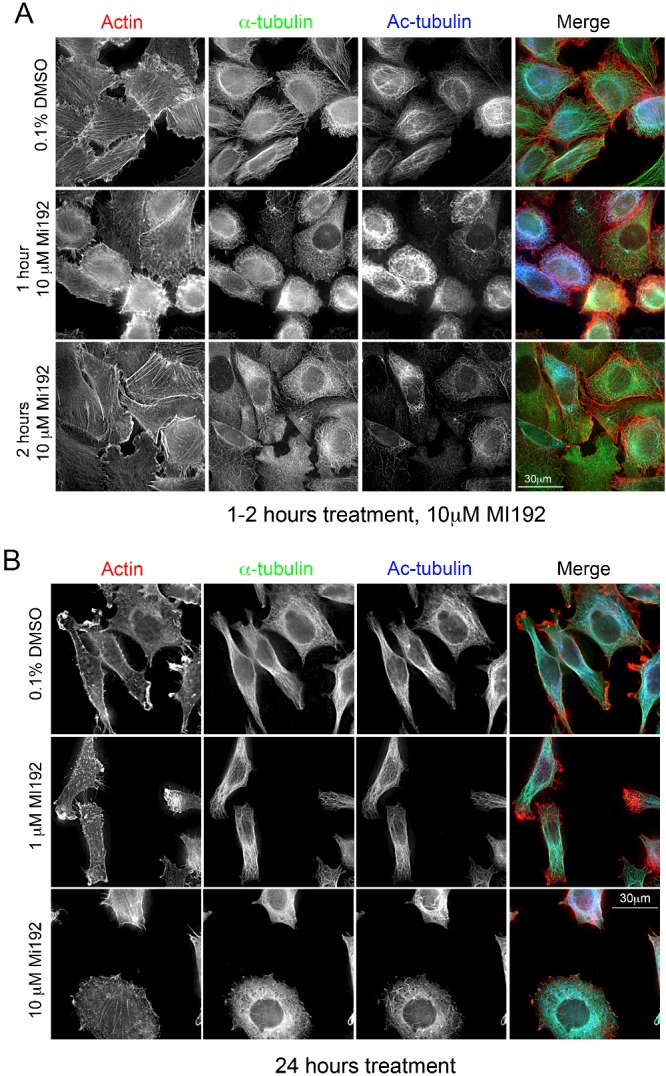
Immunostaining shows changes to acetylated tubulin organization after a short (1 h) and longer (24 h) exposure to high levels of MI192 (10 μM) (**A**) Representative images of PC3 cells stained for actin, α-tubulin and acetylated (Ac) tubulin at 1 h (control DMSO), 1 h of treatment with 10 μM MI192 and 2 h of treatment with 10 μM MI192. (**B**) Representative images of PC3 cells stained for actin (red), α-tubulin (green) and acetylated tubulin (Ac-tubulin, blue) after treatment for 24 h with 0.1% DMSO (control), 1 or 10 μM MI192.

Imaging of cells fixed and stained for actin, tubulin and acetylated tubulin, after treatment with MI192, also showed these changes in acetylated tubulin levels (Figure 2). After 1–2 h of treatment with 10 μM MI192, actin and α-tubulin expression and organization does not appear to change, but numbers of acetylated microtubules first increase (at 1 h) then decrease (at 2 h) (Figure 2A). After 24 h, we found that there were fewer microtubules present in the cells treated with 10 μM MI192, compared with cells treated with 0.1% DMSO or 1 μM MI192 (Figure 2B). Actin organization was not affected. Thus, treating PC3 cells with 10 μM MI192 initially increases levels of acetylated tubulin (within the first 1 h) and then decreases levels. Although levels of tubulin remain unaltered (by Western blotting), the rapid fall in tubulin acetylation is associated with significant tubulin depolymerization.

### MI192 significantly reduces microtubule dynamics

**Figure 3:**
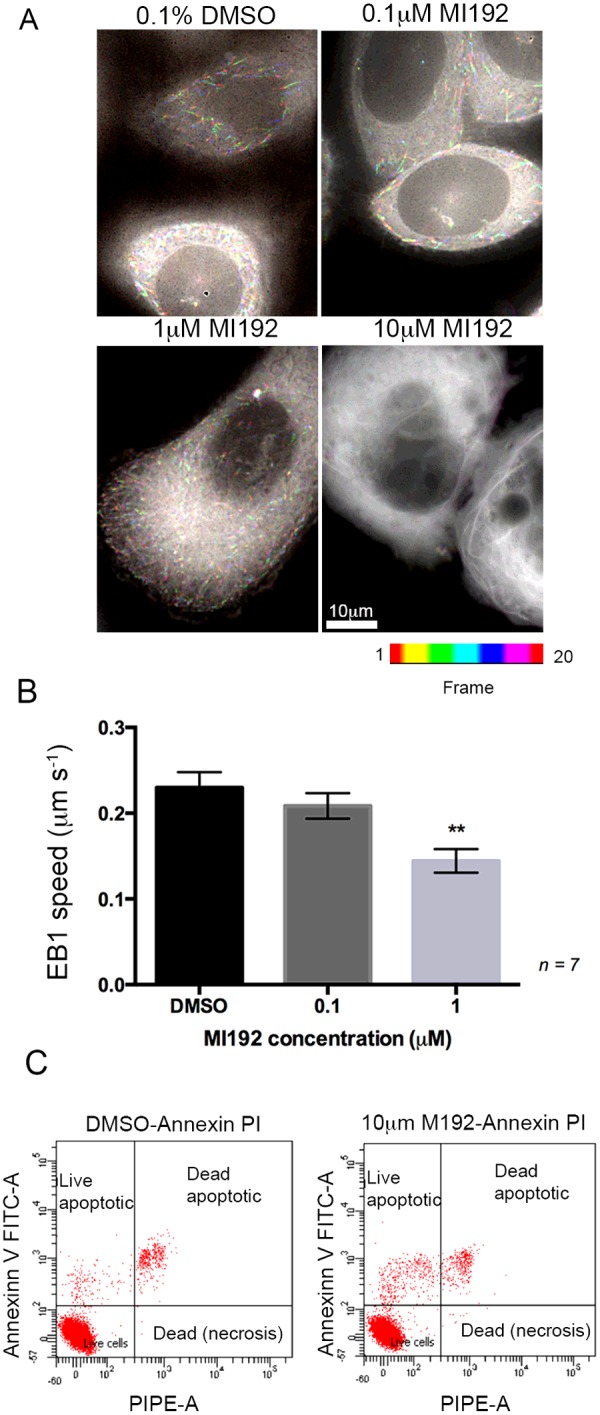
MI192 affects microtubule dynamics (**A**) Images of cells expressing eGFP–EB1, after treatment with 0.1, 1 or 10 μM MI192 for 1 h. Images were captured every 2 s, for a total of 40 s (a total of 20 frames). The images shown were generated from the image sequences using ImageJ Fiji, and the temporal-colour code plug, to show the lengths of the tracks and how they vary between the different conditions. The colour code reference is shown below the images. (**B**) Speed of EB1 spots, tracked for seven cells. Means ± S.E.M. are shown. **P< 0.01. (**C**) Flow cytometry analysis using FITC–annexin V and propidium iodide to estimate apoptosis (live and dead cells) and necrotic cells with or without 10 μM MI192 treatment.

The effects of MI192 on levels of acetylated tubulin were associated with alterations in microtubule dynamics, as assessed by monitoring the dynamics of eGFP-conjugated EB1, a protein that binds to growing microtubule tips. Microtubule dynamics were reduced in a MI192 concentration-dependent manner in cells expressing eGFP–EB1 after 1 h of treatment (Figure 3). At 10 μM MI192, no dynamic microtubule behaviour was observed, and EB1 did not preferentially bind to the microtubule tips but bound non-specifically along the microtubules (Figure 3A). As EB1 normally recognizes and preferentially binds to GTP-positive microtubule ends [38], this suggests the potential loss of GTP-positive microtubule caps in MI192-treated cells. Reducing the concentration of MI192 to 1 μM significantly reduced microtubule dynamics, although in this case eGFP–EB1 did label growing microtubule tips (Figure 3A). The speed of EB1-labelled microtubule tips in cells treated with 1 μM MI192 for 1 h was significantly slower (0.14±0.01 μm · s^−1^) compared with controls (0.23±0.02 μm · s^−1^; *P*≤ 0.01, *n*=7) (Figure 3B). At the lowest MI192 concentration tested (0.1 μM), microtubule dynamics were slightly reduced, but were not significantly different from controls (Figure 3B).

### MI192 induces pro-apoptotic mechanisms in prostate cancer cells

Given the marked effects of MI192 on tubulin, we additionally tested whether high levels of MI192 could induce apoptosis (Figure 3C). After 24 h of treatment with 10 μM MI192, we found that the proportion of live apoptotic cells increased 4-fold (from 1.6% to 6.2%) compared with controls. This was accompanied by a slight increase in the proportion of dead apoptotic cells (from 3.6% to 5.3%).

### sirna-mediated knockdown of HDAC3 increases levels of tubulin acetylation

**Figure 4:**
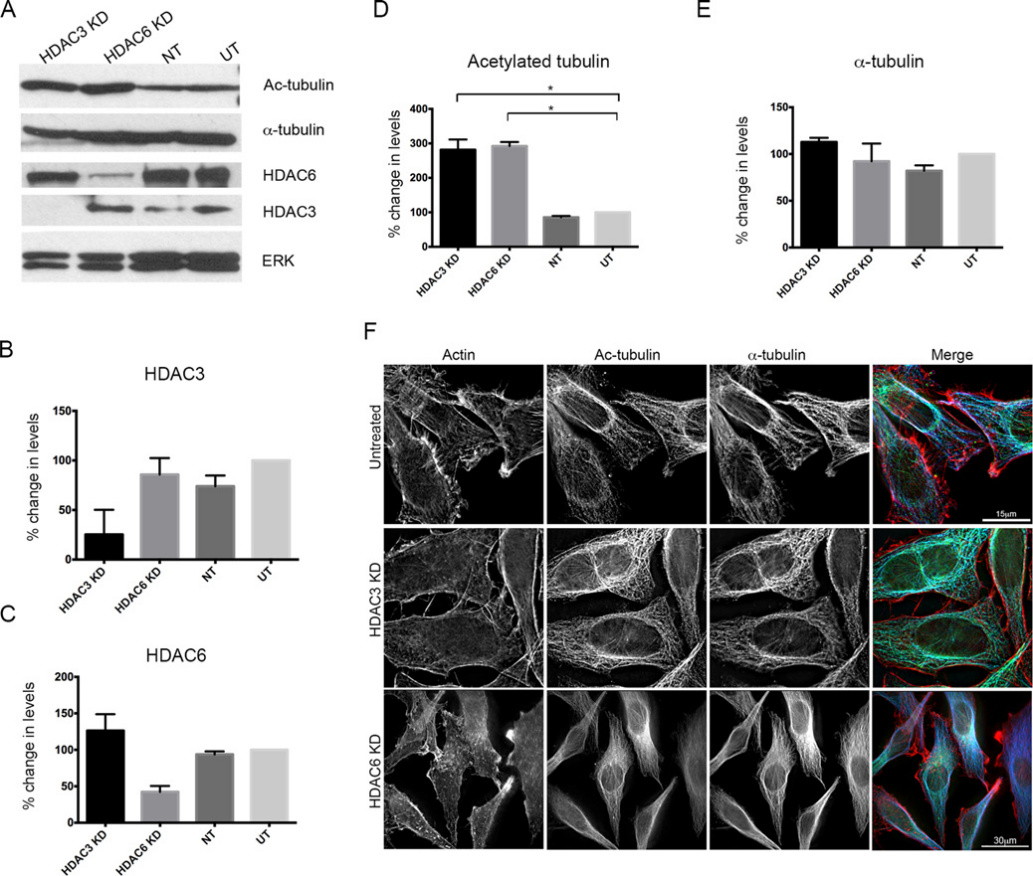
Knockdown of HDAC3 increases the levels of acetylated tubulin (**A**) Representative blots for acetylated tubulin (Ac-tubulin), α-tubulin, HDAC6 and HDAC3, and the loading control (ERK) for HDAC3 siRNA KD cells, HDAC6 siRNA KD cells, cells treated with non-targeting RNA (NT) or cells left untreated (UT). (**B**–**E**) Quantification of HDAC3 levels (**B**), HDAC6 levels (**C**), acetylated tubulin levels (**D**) and α-tubulin levels (**E**) for HDAC3 KD, HDAC6 KD, NT and UT cells. (**F**) Representative images of cells stained for actin (red in merged image), acetylated tubulin (green in merged image) and α-tubulin (blue in merged image) in UT, HDAC3 KD and HDAC6 KD cells. Means±S.E.M. are shown. *P< 0.05.

As inhibition of HDAC3 was able to modulate levels of acetylated tubulin, we next tested whether siRNA-mediated KD of HDAC3 affected tubulin acetylation. We found that levels of acetylated tubulin significantly increased when HDAC3 was depleted (Figures 4A and 4D). This increase was similar to that observed for HDAC6 KD (Figures 4A and 4D), which has been previously reported to increase tubulin acetylation [10,31]. Moreover, the siRNA pool used was specific for HDAC3 or HDAC6 respectively, as levels of HDAC6 remained unaltered when HDAC3 was depleted, and vice versa (Figures 4A–4C). Levels of α-tubulin did not change significantly (Figure 4E) as a result of either HDAC3 or HDAC6 KD. The increased levels in acetylated tubulin were also observed in cells fixed and stained for acetylated tubulin (Figure 4F).

### Overexpression of HDAC3 using eGFP–HDAC3 decreased levels of tubulin acetylation

**Figure 5:**
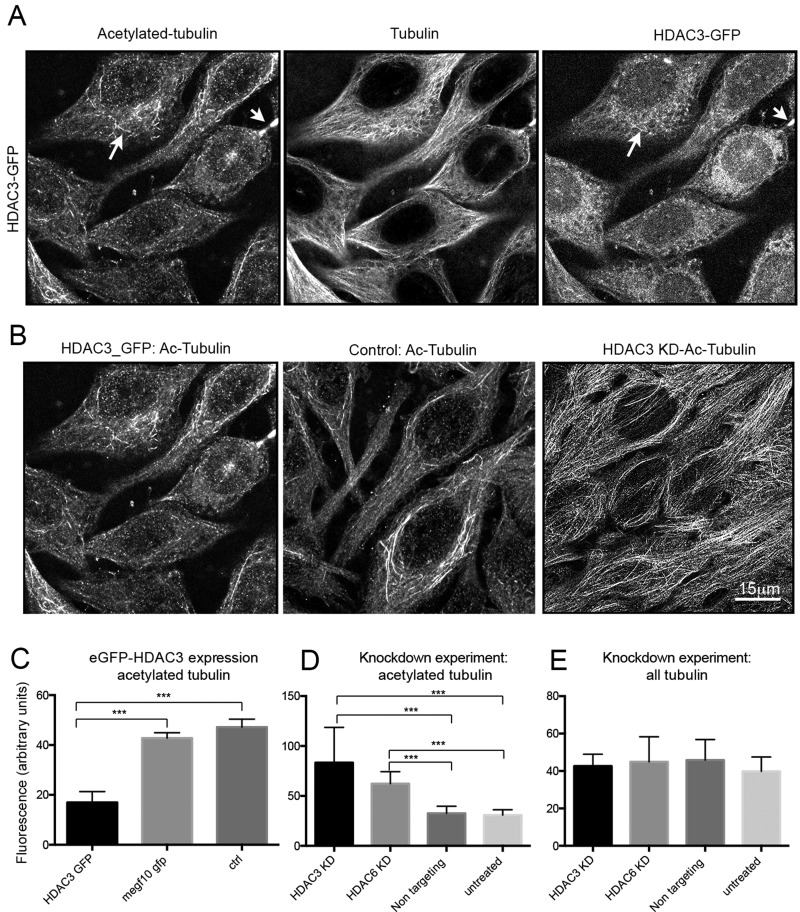
Overexpression of HDAC3 reduces levels of tubulin acetylation (**A**) Cells expressing eGFP–HDAC3, co-stained for tubulin and acetylated tubulin. Arrows indicate regions where there is some co-localization between eGFP–HDAC3 and acetylated tubulin. (**B**) Cells stained for acetylated tubulin (Ac-tubulin) in HDAC3 KD cells, control [untreated (UT) cells] and in cells expressing eGFP–HDAC3 (as in **A**). (**C** and **D**) Changes in levels of acetylated tubulin assessed from fluorescent images for eGFP–HDAC3 expression and HDAC3 KD experiments. (**E**) Assessment of total tubulin expression from fluorescent images for KD experiments. *n*=9 cells, data shown as means ± S.D. ***P< 0.001.

Cells overexpressing HDAC3 using an eGFP–HDAC3 expression adenovirus, showed reduced levels of tubulin acetylation (Figure 5A) compared with wild-type cells, as shown by immunostaining. Analysis of HDAC3–GFP localization showed that levels of GFP–HDAC3 were approximately 2-fold higher in the nucleus than in the cytoplasm. Acetylated microtubules were still present in the cytoplasm, but commonly more fragmented. Staining for α-tubulin showed that microtubules were still abundant (Figure 5A), and thus the lack of staining for acetylated tubulin is not due to a loss of microtubules overall. eGFP–HDAC3 could occasionally be found associated with microtubules in these overexpressing cells in the immunofluorescence images (Figure 5A). Directly comparing the staining pattern for acetylated tubulin between HDAC3 KD cells, wild-type and eGFP–HDAC3- expressing cells showed the abundant levels of acetylated tubulin in KD cells, compared with the fragmented appearance of acetylated tubulin in eGFP–HDAC3-expressing cells (Figure 5B). Quantification of the fluorescence levels for acetylated tubulin in HDAC3 KD and eGFP–HDAC3-overexpressing cells compared with untreated controls, or cells infected with an adenovirus expressing an unrelated eGFP-fusion protein (MEGF10–GFP) showed that levels of acetylated tubulin were significantly reduced in HDAC3-overexpressing cells compared with controls (Figure 5C), and significantly increased in HDAC3 KD cells compared with controls (Figure 5D), whereas levels of unmodified tubulin remained the same (Figure 5E).

### Exogenous expression of eGFP–HDAC3 or knockdown of HDAC3 had minor effects on mitosis

**Figure 6:**
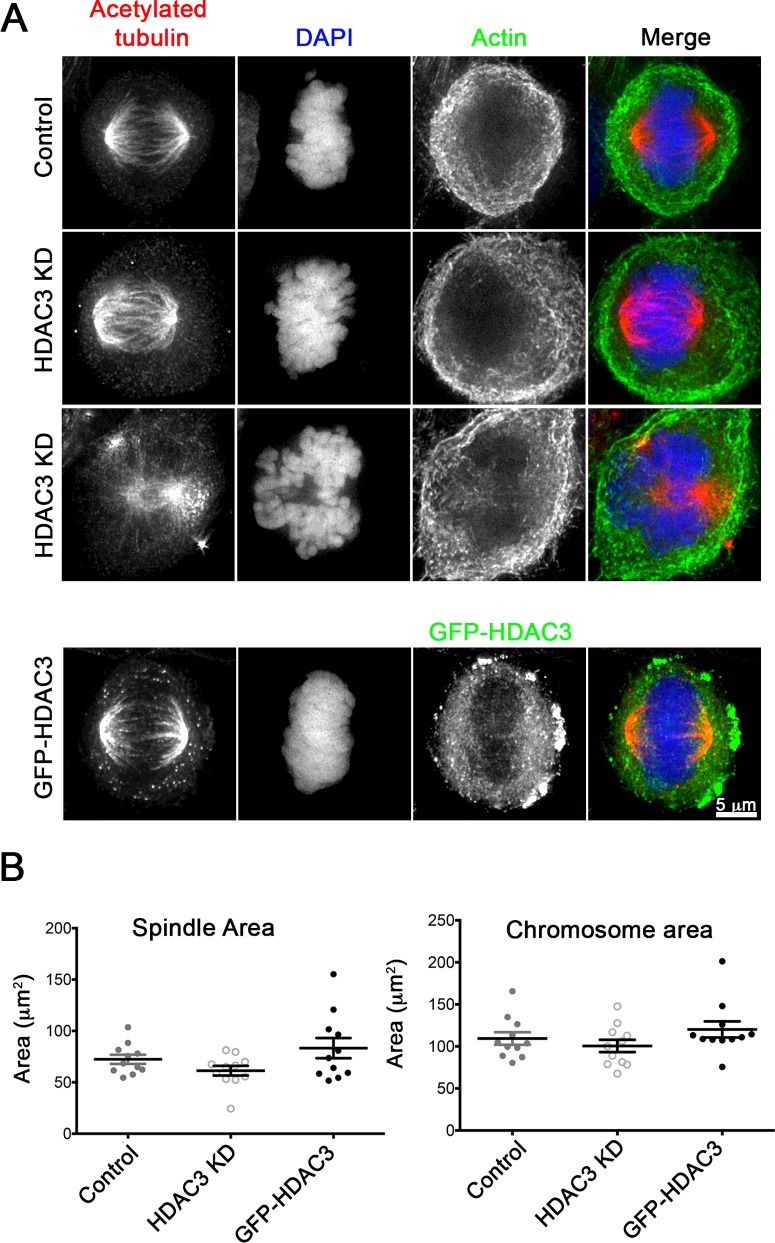
Effect of HDAC3 KD and expression of GFP–HDAC3 on mitosis (**A**) Representative images of mitotic cells for controls [treated with non-targeting (NT) siRNA], HDAC3 KD and GFP–HDAC3-expressing cells. The control and HDAC3 KD cells were stained for acetylated tubulin (red), with DAPI (blue) and for F-actin (green). Two examples are provided for HDAC3 KD cells to demonstrate that we did see some collapsed spindles as reported previously [27]. GFP–HDAC3 cells were only stained for acetylated tubulin (red) and DAPI (blue), and the image for GFP–HDAC3 is shown in green. (**B**) Quantification of spindle and chromosome areas for control, HDAC3 KD and GFP–HDAC3-expressing cells. Ten to twelve cells were imaged at high magnification and areas were quantified in imageJ. Individual results are shown with means ± S.E.M. overlaid.

We also examined the effects of expressing eGFP–HDAC3 or reducing expression levels of HDAC3 by siRNA on mitosis. Previously, it has been reported that HDAC3 is recruited to the mitotic spindle, where it is required for kinetochore–microtubule attachment, and that KD of HDAC3 reduced the width of the spindle, and could result in a ‘dome’-shaped chromosome organization [27]. We found that the metaphase spindle area was slightly reduced in KD cells, and increased in eGFP–HDAC3-expressing cells, but this difference was not significant (Figures 6A and 6B). Similar changes were observed for the area of the chromosomes. We did not find that eGFP–HDAC3 was strongly associated with the mitotic spindle in metaphase as reported previously, for metaphase spindles stained for endogenous HDAC3 [27]. Metaphase spindles did not have obvious changes in levels of acetylated tubulin between control, KD and eGFP–HDAC3-expressing cells (Figure 6A), suggesting that, whereas HDAC3 may play a role in deacetylation of microtubules in interphase cells, it may not have marked effects on acetylated tubulin levels in the metaphase spindle. We also observed a small but significant increase in the numbers of cells undergoing cytokinesis in both KD and eGFP–HDAC3-expressing cells from 1.3±0.4% of cells under control conditions, to 3.4±0.4% in KD cells and 4.9±0.6% in eGFP–HDAC3-expressing cells.

### The effect of HDAC3 on tubulin acetylation is indirect

**Figure 7:**
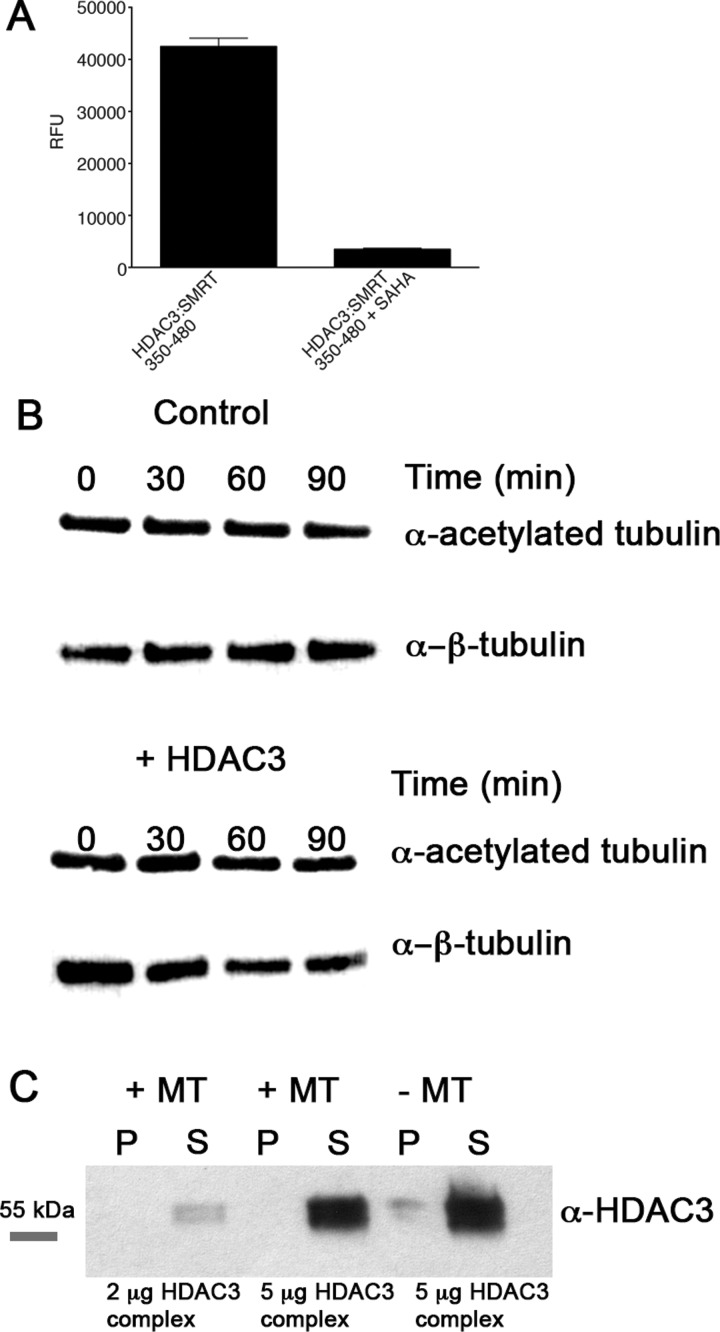
Effect of active HDAC3–SMRT–DAD complex on tubulin acetylation and tubulin association *in vitro* (**A**) The HDAC3–SMRT 350–480 complex is enzymatically active and can be readily inhibited by the HDAC inhibitor SAHA. (**B**) Representative Western blots for acetylated tubulin, and for β-tubulin (as a control for total amount of tubulin) in the absence (Control) and presence of the HDAC3 complex following incubation for different lengths of time. (**C**). Representative Western blot for a microtubule-binding assay at two different HDAC3 complex concentrations, in the presence or absence of microtubules.

Finally, we tested whether HDAC3 can directly deacetylate microtubules *in vitro*, using a purified active HDAC3–SMRT complex, purified from mammalian cells [37]. Although HDAC3 can be expressed in bacteria, it is unclear whether the purified protein is active, as it is not able to interact with SMRT [39]. The purified HDAC3–SMRT complex was first shown to be active (Figure 7A). Incubating the active complex with purified tubulin did not affect levels of tubulin acetylation (Figure 7B). Moreover, spin-down assays with tubulin–HDAC3 complexes failed to co-precipitate the HDAC3 complex with tubulin (Figure 7C). Therefore the effects on tubulin acetylation we observed in the cell following inhibition or KD of HDAC3 are likely to be through an indirect mechanism.

## DISCUSSION

Our results provide evidence that HDAC3 indirectly modulates levels of tubulin acetylation in cells. HDAC3 is widely expressed, suggesting that HDAC3 may modulate tubulin acetylation in many cells. Treatment with high levels (10 μM) of MI192, a specific inhibitor of HDAC3, showed an acute increase in acetylated tubulin after 1 h of treatment, followed by a sharp decrease up to 24 h later. The decrease in levels of acetylated tubulin levels at 24 h was associated with a reduction in polymerized microtubules. Treatment for 24 h at lower levels of MI192, close to its IC_50_ (500 nM), modestly increased levels of acetylated tubulin. siRNA-mediated KD of HDAC3 also increased levels of acetylated tubulin. In contrast, expression of eGFP–HDAC3 reduced levels, staining for acetylated tubulin showed a more highly fragmented pattern, and eGFP–HDAC3 appeared to be partly associated with acetylated microtubules. In mitosis, exogenous expression of eGFP–HDAC3 had a small effect on spindle formation and chromosomal organization, and acetylated tubulin was still observed in the spindle microtubules. *In vitro* experiments failed to show a direct association of eGFP–HDAC3 with microtubules or to show any changes in tubulin acetylation. Taken together, our results suggest that HDAC3 may play a modulatory role for tubulin acetylation particularly in interphase cells, through an indirect route.

HDAC3, unlike the two other Class I HDAC members, HDAC1 and HDAC2, is found in both the nucleus and the cytoplasm, and its cytoplasmic roles are largely unexplored. Our results suggest that it has a novel cytoplasmic role; modulation of levels of tubulin acetylation. Broad-spectrum HDAC inhibitors including TSA (which inhibits Class I HDACs [40]) and valproic acid (which inhibits Class I/II HDACs [41]) have been shown to modulate levels of tubulin acetylation, and these were suggested to mediate their effects by inhibiting HDAC6, which is well known to modulate tubulin acetylation [10,31]). However, our results with the highly selective HDAC3 inhibitor MI192 suggest that these broad-spectrum inhibitors may additionally inhibit the deacetylation of tubulin by HDAC3. The exact mechanism by which MI192-mediated inhibition of HDAC3 results in an acute 4-fold increase in tubulin acetylation followed by a rapid reduction in in tubulin acetylation levels, is unclear. However, siRNA KD of HDAC3 also increased levels of tubulin acetylation, and this increase was similar to that resulting from siRNA-mediated KD of HDAC6, which is well established as being able to deacetylate tubulin [10,31]. Moreover, overexpression of HDAC3 reduced tubulin acetylation. Thus, it seems likely that HDAC3 is involved in modulating tubulin acetylation in cells, although the exact mechanism by which it does so now needs to be established, as our *in vitro* experiments suggest that an active HDAC3 complex is unable to do this directly.

The novel benzamide derivative compound MI192 has previously been shown to act as a potent and selective Class I HDAC inhibitor, with slower on/off binding kinetics (and thus longer-lasting effects), and greater overall activity than related inhibitors [30]. Its measured IC_50_ in HeLa cells was higher (1.5 μM [30]) than measured here in PC3 cells (450 nM; total HDAC activity) suggesting that MI192 may have a slightly increased potency in PC3 cells. However, this will depend on the total levels of HDAC3 in PC3 compared with HeLa cells. It is worth noting that elevated HDAC3 levels are a common hallmark of tumour cells. Gliomas [42] and colon cancer cells [43] have both been shown to have elevated levels of HDAC3. An analysis of a wide range of human cancers showed that HDAC3 was expressed at high levels in many cancerous tissues and cell lines, including PC3 cells [44]. A further study shows that HDAC3 is strongly expressed in over 90% of prostate cancer samples tested [45]. Thus MI192 has potential for use as a therapeutic in prostate cancer through its ability to affect microtubule acetylation, polymerization and dynamics.

As well as microtubule depolymerization, the reduction in acetylated tubulin induced by MI192 also appeared to stimulate apoptosis. MI192 was shown to induce apoptosis in leukaemia cell lines, although the effect was variable among the three cell lines tested [30]. MI192 may activate a conserved pathway for apoptosis in multiple cancer cell lines. The tumour-suppressor gene p53, one of the most commonly mutated genes in cancer cells, is important for stimulating apoptosis. Post-translational acetylation of p53 up-regulates its activity [46], and HDAC1, HDAC2 and HDAC3 are all capable of down-regulating p53 activity by deacetylation [47]. However, this is unlikely to be the explanation for the effect of MI192 in our experiments, as PC3 cells do not express p53 [48].

HDAC3 was previously reported to localize to the mitotic spindle in prophase but not in metaphase, in HeLa cells, HEK-293 cells and mouse 3T3 fibroblasts, and KD of HDAC3 was reported to reduce the width of the spindle [27]. In our experiments, we also found some spindles that were smaller, and collapsed in the HDAC3 KD cells and chromosomal organization in those cells was aberrant. We also found that the spindle size increased in eGFP–HDAC3-expressing cells, and that cytokinesis was apparently delayed in both HDAC3 KD and eGFP–HDAC3-expressing cells. The cytoplasmic bridge connecting the two daughter cells is rich in acetylated tubulin, although it is still unclear why this is the case. Acetylation of tubulin does not apparently change its structure [49]. One suggestion is that acetylated tubulin may be important for new membrane formation during cytokinesis [50]. Thus, altering tubulin acetylation levels by KD or overexpression of HDAC3 might be expected to delay cytokinesis. In contrast with our results, KD of HDAC3 did not apparently alter levels of tubulin acetylation in a previous report [27], but this report focused on mitosis only and did not show images for microtubules in interphase cells. Moreover, they were unable to determine the exact mechanism by which HDAC3 had effects on spindle organization in metaphase.

In conclusion, our data using a targeted inhibitor (MI192) for HDAC3 and complimentary HDAC3 KD experiments suggest that HDAC3 is involved in regulating tubulin acetylation levels, as one of its cytoplasmic roles, through an indirect route. Moreover, the inhibitor MI192 may exert some of its phenotypic effects on cells through this mechanism.

## Abbreviations

DAD: deacetylase-activating domain
EB1: end-binding 1
ERK: extracellular-signal-regulated kinase
HDAC: histone deacetylase
HEK: human embryonic kidney
IκBα: inhibitor of NF-κB α
IL: interleukin
KD: knockdown
NCoR: nuclear receptor co-repressor
NF-κB: nuclear factor-κB
SAHA: suberoylanilide hydroxamic acid
SMRT: silencing mediator of retinoic and thyroid receptors
STAT: signal transducer and activator of transcription
TBL1: transducing-β-like protein 1
TNF: tumour necrosis factor
